# The role of immune checkpoint inhibitors in the first-line treatment for patients with advanced biliary tract cancer: a systematic review and meta-analysis of randomized trials

**DOI:** 10.3389/fonc.2024.1409132

**Published:** 2024-07-18

**Authors:** Elsa Vitale, Alessandro Rizzo, Lorenza Maistrello, Patrizia Nardulli, Tiziana Talienti, Davide Quaresmini, Simona De Summa, Raffaella Massafra, Nicola Silvestris, Oronzo Brunetti

**Affiliations:** ^1^ Department of Mental Health, Bari Local Health Authority, Scientific Directorate, IRCCS Istituto Tumori “Giovanni Paolo II”, Bari, Italy; ^2^ IRCCS Istituto Tumori “Giovanni Paolo II”, Bari, Italy; ^3^ San Camillo Hospital (IRCCS), Venice, Italy; ^4^ University of Messina, Messina, Italy

**Keywords:** cholangiocarcinoma, biliary tract cancer, durvalumab, immunotherapy, pembrolizumab, immune checkpoint inhibitors

## Abstract

**Background:**

We performed a systematic review and meta-analysis to further explore the impact of the addition of immunotherapy to gemcitabine–cisplatin as first-line treatment for advanced biliary tract cancer (BTC) patients.

**Methods:**

Literature research was performed, and hazard ratio values and 95% confidence intervals were calculated. Heterogeneity among studies was assessed using the tau-squared estimator $\left(\tau^{2}\right)$ . The total Cochrane Q test (Q) was also assessed. The overall survival rate, objective response rate, and progression-free survival in the selected studies were assessed.

**Results:**

A total of 1,754 participants were included. Heterogeneity among the studies selected was found to be non-significant (p = 0.78; tau^2^ = 0, I^2^ = 0%). The model estimation results and the forest plot suggested that the test for the overall effect was significant (Z = −3.51; p< 0.01).

**Conclusion:**

The results of the current meta-analysis further confirm the role of immune checkpoint inhibitors plus gemcitabine–cisplatin as the new standard first-line treatment for advanced BTC patients.

**Systematic review registration:**

https://www.crd.york.ac.uk/prospero, identifier CRD42023488095.

## 1 Introduction

Biliary tract cancer (BTC) includes several malignancies in both the intrahepatic or extrahepatic bile ducts and gallbladder (1); despite this cancer records, there is an increased number of cases worldwide with an annual incidence varying from 0.72 to 1.62 per 100,000 individuals according to different countries over the last 30 years (2, 3).

Unfortunately, only 20% of BTC patients may be eligible for radical resection (4). Due to late-stage diagnosis, the 5-year overall survival (OS) rate of patients with locally advanced, unresectable, and metastatic disease is very low, and for more than a decade, the most effective first-line treatment option was considered the combination of gemcitabine plus cisplatin (5, 6), following the results of the practice-changing ABC-02 study published by Valle et al. (7). This phase III trial reported a median OS of 11.7 months (95% CI 9.5–14.3) for gemcitabine plus cisplatin compared to 8.1 months (7.1–8.7) for gemcitabine alone [hazard ratio (HR) 0.64, p< 0.001]. Additionally, in patients experiencing disease progression following gemcitabine–cisplatin, fluorouracil-based combinations are among the standard second-line options; however, the efficacy of these treatments is overall low, with short median progression-free survival (PFS) (8, 9).

Recent years have unveiled the biological and molecular landscape of these tumors by including specific molecular alterations in which targeted systemic therapies have improved the clinical outcomes of metastatic patients (10, 11). This option represents a therapeutic niche given the relatively low incidence of these genetic alterations, according to different tumor sites (12).

Immune checkpoint inhibitors have drastically extended the survival and quality of life of cancer patients over the last 10 years (13). In fact, the efficacy of inhibitors or antibodies targeting immune checkpoints, such as programmed cell death ligand 1 (PD-L1), programmed death protein 1 (PD-1), cytokine T-lymphocyte-associated protein 4 (CTLA-4), TIM-3, lymphocyte activation gene 3 protein (LAG-3), and TIGIT, has been tested in several hematological and solid tumors. In BTC patients, the randomized, double-blind, phase III TOPAZ-1 study reported that the addition of the PD-L1 inhibitor durvalumab to the standard first-line doublet gemcitabine–cisplatin was associated with a statistically significant and clinically meaningful increase in terms of median OS compared to gemcitabine plus cisplatin alone (12.8 months [95% CI 11.1–14.0] in the durvalumab group *vs*. 11.5 months [10.1–12.5] in the placebo group; HR 0.80 [95% CI 0.66–0.97]; two-sided p = 0.021) (14). More recently, the PD-1 inhibitor pembrolizumab has been tested as first-line treatment in combination with gemcitabine–cisplatin in the KEYNOTE-966 clinical trial comparing the immune-based combination with gemcitabine–cisplatin alone as front-line treatment (14, 15). This phase III study evidenced a significant improvement in the survival rate for the experimental arm (15), something that supports the role of pembrolizumab combined with gemcitabine and cisplatin as first-line therapy for unresectable BTC patients.

Based on these premises, the current meta-analysis aimed to further investigate the role and the clinical impact of the addition of immune checkpoint inhibitors to gemcitabine and cisplatin as first-line treatment in BTC patients.

## 2 Materials and methods

**Figure 1:**
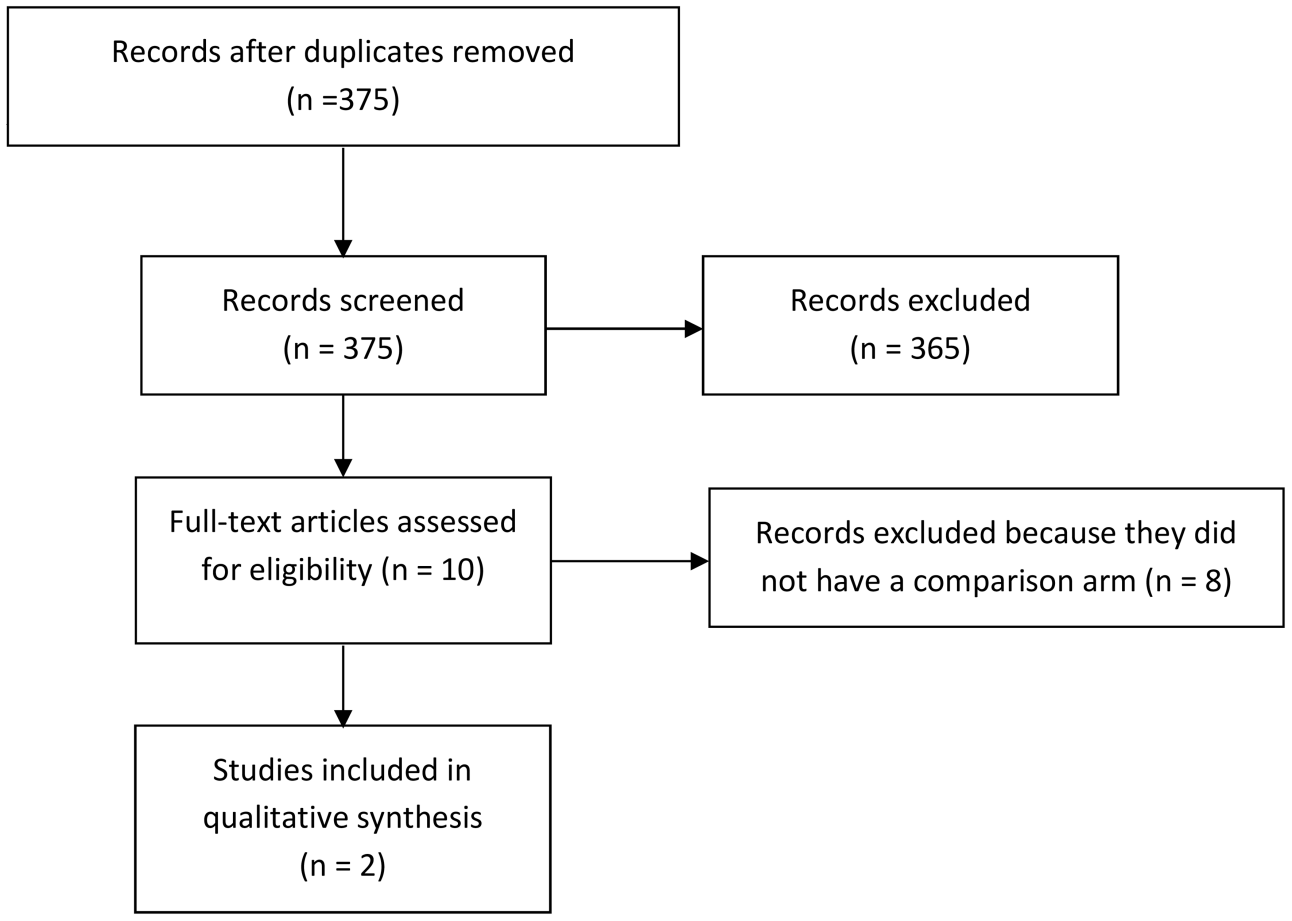
PRISMA flow diagram adapted to the present meta-analysis. PRISMA, Preferred Reporting Items for Systematic Reviews and Meta-Analyses.

The present systematic review and meta-analysis was recorded in the PROSPERO register with register no. CRD42023488095. By considering the Preferred Reporting Items for Systematic Reviews and Meta-Analyses (PRISMA) flowchart (16) (**Figure 1**), a systematic literature review was carried out using the Embase, PubMed, Scopus, and Web of Science databases. Keywords included immune checkpoint inhibitors (ICI) OR immunotherapy AND chemotherapy (CT), OR combination of CT with immune checkpoint inhibitors (ICIs-CT), AND biliary tract cancer OR cholangiocarcinoma.

### 2.1 Inclusion criteria

The present review includes all phase III clinical trials enrolling BTC adults (>18 years) and comparing first-line immune checkpoint inhibitors plus gemcitabine–cisplatin with gemcitabine–cisplatin alone for the treatment of BTC.

### 2.2 Data extraction (selection and coding)

Initially, records were identified through a systematic database search and were uploaded. Then, duplicate studies were removed. Three independent reviewers (O.B., A.R., and E.V.) assessed the title and abstract of the identified studies for inclusion, and unsuitable reports were removed. After that, articles were uploaded, and the full texts were evaluated more closely for eligibility. Disagreements about whether a study should be included or not were resolved by discussion and consensus. If the disagreement remained, arbitration from another reviewer was provided (D.Q.). Data collection was extracted by considering study characteristics (author, year of publication, aim, design, sample size, and setting), participants (age, biliary tract cancer stage, and type of treatments performed), and outcome in overall survival and a manageable safety profile in BTC patients.

### 2.3 Type out outcome measures

We examined a total of 375 articles. Then, we excluded 365 articles that did not fill our inclusion criteria. From the remaining 10 articles, we removed the other eight articles since they did not have a comparison arm without extraction data from the safety analysis. Finally, two different authors extracted data from the safety analysis (**Figure 1**). We obtained data from the full text of each study. Additionally, we assessed the OS as the percentage of BTC patients who are still alive for a certain period of time after they were diagnosed with or started treatment, the overall response rate (ORR) as the percentage of people in a study or treatment group who have a partial response or complete response to the treatment within a certain period, and the PFS as the length of time during and after the treatment of a disease, such as cancer.

### 2.4 Quality assessment

The articles selected were assessed according to the Joanna Briggs Institute through the Checklist for Randomized Controlled Trials (17) by independently involving the first and second authors, and eventual disagreements were resolved by consensus or with the involvement of the third and fourth authors, if necessary. The Checklist for Randomized Controlled Trials included a total of 13 assessment items, for which one of the authors should give a judgment of total clarity/completeness (yes) or, conversely, total confusion (no) or a judgment of poor clarity (unclear) or, finally, not appreciable judgment (N/A) (**Table 1**).

**Table 1:** Quality assessment of the selected studies.

Quality assessment/items	Selected studies/authors’ judgments	
	14	15
**Item no. 1:** Was true randomization used for assignment of participants to treatment groups?	Y	Y
**Item no. 2:** Was allocation to treatment groups concealed?	Y	Y
**Item no. 3:** Were treatment groups similar at the baseline?	Y	Y
**Item no. 4:** Were participants blind to treatment assignment?	Y	Y
**Item no. 5:** Were those delivering treatment blind to treatment assignment?	Y	Y
**Item no. 6:** Were outcome assessors blind to treatment assignment?	U	Y
**Item no. 7:** Were treatment groups treated identically other than the intervention of interest?	Y	Y
**Item no. 8:** Was follow-up complete and if not, were differences between groups in terms of their follow-up adequately described and analyzed?	Y	Y
**Item no. 9:** Were participants analyzed in the groups to which they were randomized?	Y	Y
**Item no. 10:** Were outcomes measured in the same way for treatment groups?	Y	Y
**Item no. 11:** Were outcomes measured in a reliable way?	Y	Y
**Item no. 12:** Was appropriate statistical analysis used?	Y	Y
**Item no. 13:** Was the trial design appropriate, and any deviations from the standard RCT design (individual randomization, parallel groups) accounted for in the conduct and analysis of the trial?	Y	Y

Y, yes; N, no; U, unclear; NA, not applicable; RCT, randomized controlled trial.

### 2.5 Measures of effect

From the individual studies, their HR values and 95% confidence intervals (95% CIs) were calculated. Then, aggregate estimates of the logarithm of the HR [ln(HR)] and 95% CI were assessed using the inverse variance method (18). Heterogeneity among studies was assessed using the tau-squared estimator $\left(\tau^{2}\right)$ by evaluating the Paule–Mandel method (19), which estimated the variance of the distribution of true effect sizes. Additionally, the total Cochrane Q test (Q) was assessed to indicate any lack of homogeneity of the results of the included studies. Finally, the Higgins and Thompson I-square inconsistency index (*I* ^2^) (20) was adopted whose values were classified as low (25%–50%), moderate (50%–75%), or high (75%) (20).

Due to the low number of studies, data were pooled using a meta-analytic method based on a fixed-effects model (21–23).

Forest plots were generated for each meta-analysis (24). Publication bias was assessed by visually performing and inspecting a contour-enhanced funnel plot.

The analyses were performed using R Studio v.4.3.1 software (25, 26), and the significance level was set at 0.05.

## 3 Results

A total of 1,754 patients were enrolled in the two studies selected from this systematic meta-analysis. Specifically, 685 BTC patients were enrolled in the TOPAZ-1 trial and assessed durvalumab plus chemotherapy (14). Of these, 341 belonged to the experimental group and reported a median age of 64 years (20–80), and 344 patients belonged to the control group with a median age of 64 years (31–85). However, the KEYNOTE-966 study assessed the pembrolizumab effects plus chemotherapy in BTC patients (15). A total of 1,096 patients were involved: 533 belonged to the experimental group and 536 to the control group. Of the participants, 50% of the experimental group and 56% of the control group were aged less than 65 years. **Table 2** shows the site of origin, highlighting differences among BTC sub-clinical specializations assessed in the two studies included in the meta-analysis.

**Table 2:** Site of origin compared within the two studies selected.

Site of origin/studies included	Durvalumab protocol (14)		Pembrolizumab protocol (15)	
	Exp. group (n = 341) n (%)	CTRL (n = 344) n (%)	Exp. group (n = 533) n (%)	CTRL (n = 536) n (%)
**Extrahepatic**	66 (19.4)	65 (18.9)	98 (18)	105 (20)
**Gallbladder**	85 (24.9)	86 (25)	115 (22)	118 (22)
**Intrahepatic**	190 (55.7)	193 (56.1)	320 (60)	313 (58)

**Figure 2:**
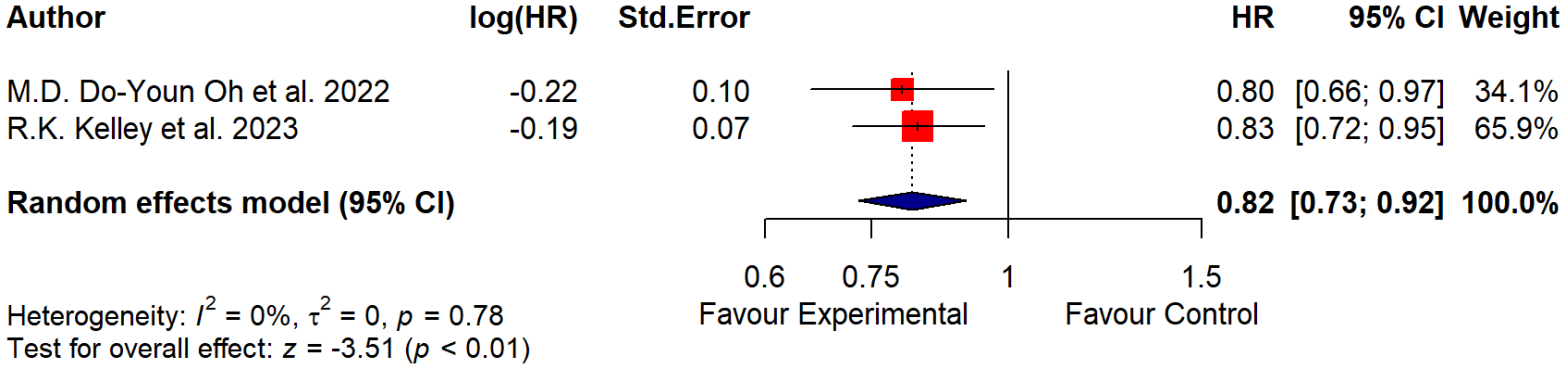
Forest plot of the treatment effect.

Data from two studies (14, 15) with 1,754 participants were pooled. Heterogeneity among the studies selected was found to be non-significant (p = 0.78; tau^2^ = 0 and I^2^ = 0%). The model estimation results and the forest plot (**Figure 2**) suggested that the test for the overall effect was significant (Z = −3.51; p< 0.01), so there was evidence in favor of the experimental treatment.

The squares on the graph represent the estimate of the effect for each study, while the horizontal lines that intersect the squares represent their 95% CI. The area of each square is proportional to the weight of the study and is inversely proportional to the variance of the single estimate. The diamond represents the combined estimate: the center indicates the precise and overall estimate of the effect, while the width of the sides represents the confidence interval. The vertical line (HR = 1.0) represents the statistical non-significance, which indicates the absence of detectable differences in the effects of the exposure compared. If the confidence interval of the studies crosses the vertical line, the study results should be considered not statistically significant.

**Figure 3:**
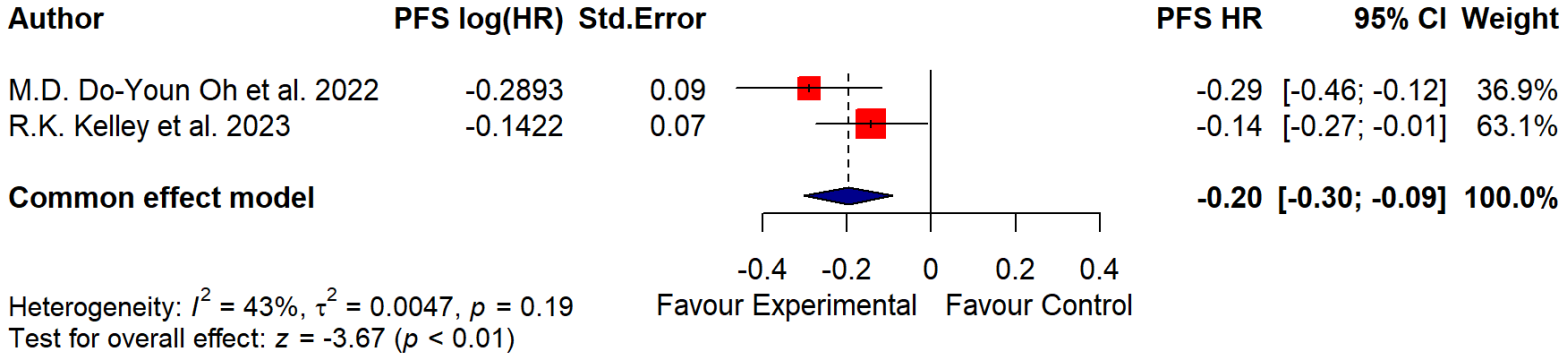
Forest plot of the PFS data.

The funnel plot was symmetrical (**Figure 3**), and all studies fell within the regions of statistical significance by also reducing the plausibility of publication bias.

The trim-and-fill method was applied to identify asymmetry and assess potential bias. The colors of the funnels indicate the respective significance levels. The white area, however, represents the area of no significance (p > 0.1) for which publication bias is a plausible explanation. In the case when there is no publication bias, all studies would lie symmetrically around our pooled effect size (the striped line) within the form of the funnel.

By considering the ORR in the two selected studies, we highlighted the ORRs in **Table 3**.

**Table 3:** The ORRs in the two selected studies.

	Do-Youn 14		15	
	Durvalumab plus gemcitabine and cisplatin (n = 341)	Placebo plus gemcitabine and cisplatin (n = 343)^†^	Pembrolizumab plus gemcitabine and cisplatin group (n = 533)	Placebo plus gemcitabine and cisplatin group (n = 536)
Objective response rate, n (%)	91 (26.7%)	64 (18.7%)	156 (29%)	152 (28%)
Complete response	7 (2.1%)	2 (0.6%)	14 (3%)	9 (2%)
Partial response	84 (24.6%)	62 (18.1%)	142 (27%)	143 (27%)

ORR, overall response rate.

† Do-Youn 14: The objective response rate analysis was based on patients in the final analysis group who had measurable disease at baseline. There was one patient in the placebo group who had no measurable disease at baseline.

In the study of Oh et al. (14), ORR was reported as 26.7% for patients (n = 91) treated with durvalumab and 18.7% (n = 64) for those who received placebo. Specifically, the number of patients with a complete response rate was seven (2.1%) with durvalumab and two (0.6%) with placebo, while the number of patients who achieved a confirmed partial response was 84 (24.6%) with durvalumab and 62 (18.1%) with placebo.

In the study of Kelley et al. (15), at the final analysis, 156 (29%) of the 533 participants in the pembrolizumab group and 152 (28%) of the 536 participants in the placebo group had a complete or partial response. Specifically, in the pembrolizumab group, 14 people (3%) received a complete response and (142 (27%) a partial response. In contrast, in the placebo group, nine people (2%) received a complete response and 143 (27%) a partial response.

By considering the two studies together, we highlighted the ORRs in **Table 4**.

**Table 4:** ORRs between the two studies selected.

	Drug (n = 874)	Placebo (n = 879)
ORR, n (%)	247 (28%)	216 (25%)
Complete response	21 (2%)	11 (1%)
Partial response	226 (26%)	205 (23%)

ORR, overall response rate.

**Figure 4:**
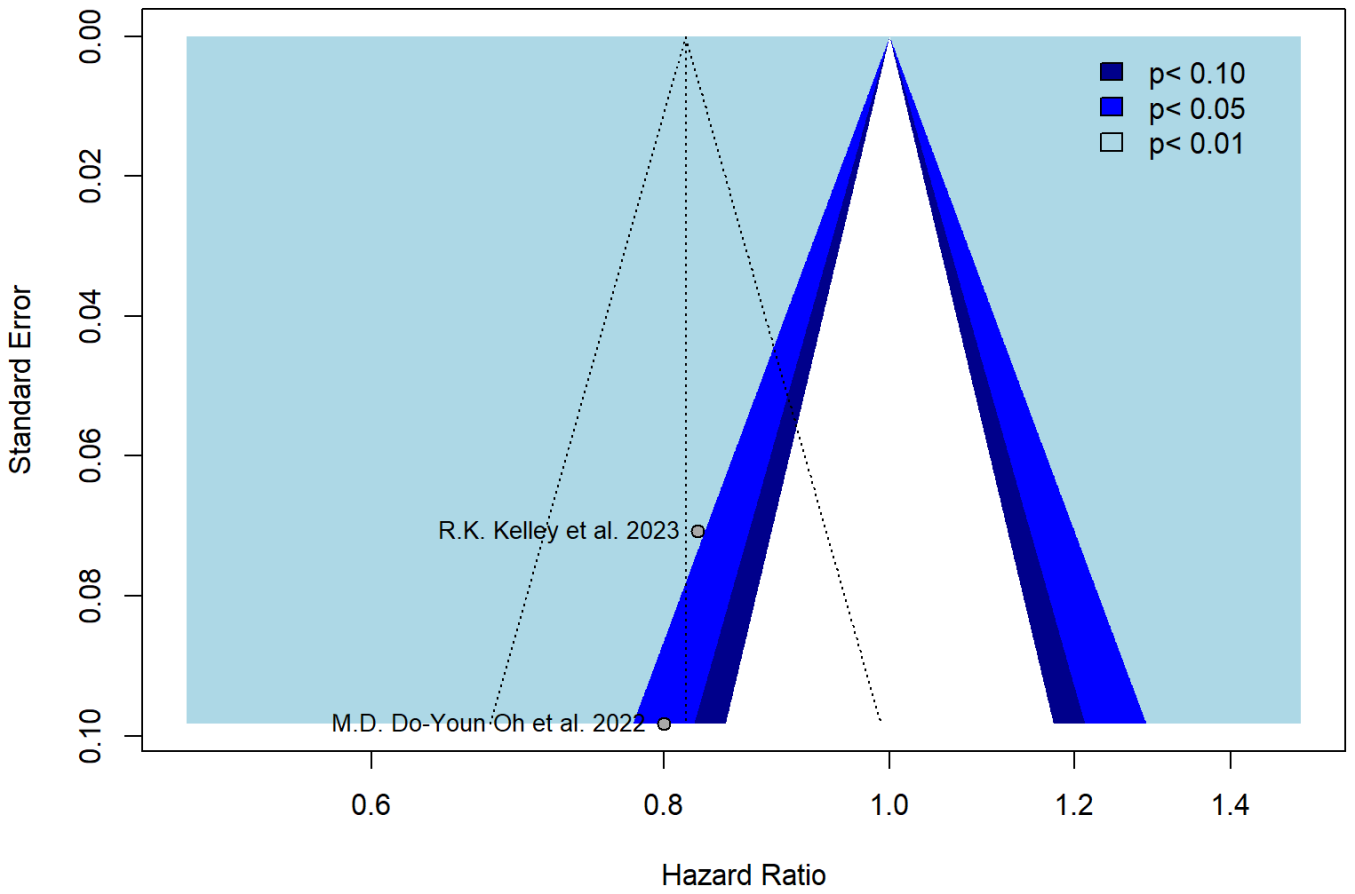
Funnel plot of the PFS data.

As regards the PFS data, which considered the time from randomization or treatment starting to the occurrence of disease progression or death, further differences were additionally highlighted in **Table 5** and **Figure 4**.

**Table 5:** The PFS in the studies selected.

	Do-Youn 14		15	
	Durva + Gem + Cis (n = 341)	Placebo + Gem + Cis (n = 344)	Pembrolizumab + gemcitabine and cisplatin (n = 533	Placebo plus gemcitabine and cisplatin (n = 536)
Median PFS, months (95% CI)	7.2 (6.7–7.4)	5.7 (5.6–6.7)	6.5 (5.7–6.9)	5.6 (4.9–6.5)
Hazard ratio (95% CI)	0.75 (0.63–0.89)		0.87 (0.76–0.99)	
Stratified log-rank p-value	p = 0.001		p< 0.05	

PFS, progression-free survival.

In the study of Oh et al. (14), the median PFS was assessed at 7.2 months in the treatment group and 5.7 in the placebo group; in the study of Kelley et al. (15), the median PFS was evaluated at 6.5 months for the treatment group and 5.6 months for the placebo group.

## 4 Discussion

In the current systematic review and meta-analysis, we investigated the role of immunotherapy combined with chemotherapy (cisplatin plus gemcitabine) compared to the standard doublet alone in the first-line therapy for advanced BTC patients included in randomized clinical trials.

Despite that preclinical reports on BTC have evidenced the presence of chronic inflammation and the improved expression of immune checkpoints, especially of PD-L1 and CTLA-4 (27, 28), early clinical trials reported low response rates and disappointing results with immune checkpoint inhibitor monotherapy in this setting (10, 29). For example, low response to PD-1 and PD-L1 inhibitors was reported by Doki et al. (30) with a median PFS of 1.5 months (95% CI 1.4– 2.6) in patients with BTC, as well as by Kim et al. (31), with a median PFS of 4.0 months (95% CI 2.3–7.6 months) in those treated with nivolumab. In addition, the lack of reliable predictors of response to immune checkpoint inhibitors is represented and still is a crucial issue in this setting (32). In order to play a synergistic role, gemcitabine and cisplatin were associated with immune checkpoint inhibitors to modulate the tumor microenvironment due to the downregulation of the immunosuppressive microenvironment and improved immunogenicity (33), as already reported in several other tumor types where immunotherapy has revolutionized clinical outcomes.

Based on these premises and following the promising evidence reported in preclinical studies (34, 35), the randomized, double-blind, phase III TOPAZ-1 study showed that combining the PD-L1 inhibitor durvalumab with gemcitabine and cisplatin led to significant improvements in terms of OS, PFS, and ORR (14). In particular, the combinatorial treatment reported a statistically significant increase in OS compared with the administration of gemcitabine–cisplatin alone (median OS 12.8 months [95% CI 11.1–14.0] in the durvalumab group *vs*. 11.5 months [10.1–12.5] in the placebo group; HR 0.80 [95% CI 0.66–0.97]; two-sided p = 0.021) (14). These results suggested the clinical value of the addition of immunotherapy to cytotoxic chemotherapy for BTC patients and represented a historical step forward. Moreover, according to TOPAZ-1, 2-year OS was 24.9% versus 10.4% of gemcitabine–cisplatin alone reported in the historical ABC-02 study; thus, these findings highlighted that exposure to immunotherapy could point toward a long-term survival and delayed clinical effects in this setting.

More recently, in the randomized, placebo-controlled, phase III KEYNOTE-966 trial, first-line pembrolizumab plus gemcitabine–cisplatin recorded significant improvement in OS rate compared with gemcitabine and cisplatin alone for BTC patients with metastatic disease (15) by observing a longer median duration of response in the pembrolizumab group than in the placebo group (9.7 months *vs*. 6.9 months, respectively).

In KEYNOTE-966 study, the efficacy boundary for a statistically significant OS benefit for the pembrolizumab group was registered, also considering BTC according to their sites of origin, such as extrahepatic cholangiocarcinoma (HR 0.99 [95% CI 0.73–1.35]), gallbladder cancer (HR 0.96 [95% CI 0.73–1.26]), and intrahepatic cholangiocarcinoma (HR 0.76 [95% CI 0.64–0.91]) (15). Also in TOPAZ-1 (14), the OS and PFS benefits observed with durvalumab in combination with gemcitabine and cisplatin were generally consistent according to their sites of origin, such as extrahepatic cholangiocarcinoma (HR 0.76 [95% CI 0.49–1.19]), gallbladder cancer (HR 0.94 [95% CI 0.65–1.37]), and intrahepatic forms (HR 0.76 [95% CI 0.58–0.98]).

Considering the evidence from the existing literature, we aimed to perform a meta-analysis focusing on the role of immunotherapy by selecting scientific papers according to the PRISMA methodology (14–16).

Moreover, the present meta-analysis considered only two phase III randomized studies available in the current literature (14, 15), which seemed to have no significant publication bias by lying symmetrically around their pooled effect size (p > 0.1).

At the same time, important differences in terms of study design should be considered. The results of KEYNOTE-966 certainly complete those of TOPAZ-1, with the former study including a larger patient population (1,069 versus 685) and a larger prevalence of non-Asian patients (55% versus 45%) and hepatitis B virus (HBV)-positive patients (30% versus 20%). In addition, while in TOPAZ-1 gemcitabine was limited to eight cycles, in KEYNOTE-966, gemcitabine chemotherapy was administered until disease progression or unacceptable toxicity, without a fixed number of administrations. In this aspect, the dissimilar number of gemcitabine administrations in KEYNOTE-966 and TOPAZ-1 may have introduced a bias and may suggest an essential diversity in clinical practice, which supply different data collection without a recognizable standard of care all around the world (14, 15). In this regard, it would be interesting to further understand the role of gemcitabine maintenance in clinical practice in real-world studies.

In a multicentric retrospective real-world study, Rimini et al. (36) confirmed the role of the combination of durvalumab with standard chemotherapy, as gemcitabine plus cisplatin, by considering it as the first-line treatment for advanced BTC. According to the present findings, the addition of immune checkpoint inhibitors to gemcitabine–cisplatin reported a statistically significant and clinically meaningful improvement in terms of clinical outcomes. These results are particularly important and interesting in a scenario where the poor prognosis and the high prevalence of cases inevitably require more effective treatment options.

However, the impact of adverse effects in the intervention treatment in both the studies included in this meta-analysis should be considered. In fact, in the study of Kelley et al. (15), adverse events caused death in 31 (6%) participants in the pembrolizumab group and 49 (9%) participants in the placebo group, which led to disruption of one or more study drugs in 138 (26%) participants in the pembrolizumab group and 122 (23%) participants in the placebo group; discontinuation of all study drugs occurred in 35 (7%) participants in the pembrolizumab group and 39 (7%) in the placebo group. Similarly, in TOPAZ-1 (14), the number of deaths due to adverse events was 12 (3.6%) in the durvalumab group and 14 (4.1%) in the placebo group. At the same time, the rates of adverse event grades seemed to be very similar between treatment groups.

In the two studies considered in this meta-analysis, there was an essential bias in the microsatellite instability (MSI) status, which could change in all patients, and more cases were also unknown (14, 15). Therefore, data may be solely attributed to efficacy with immunotherapy in the small groups of patients with MSI or with the positive presence of predictor of immune response whose validation for BTC still needs additional data (14, 15).

In conclusion, this meta-analysis further confirmed the practice-changing role of immunotherapy in combination with standard chemotherapy (gemcitabine plus cisplatin) compared to standard treatment alone. Immunotherapy clinically improved OS and PFS rates in the BTC population; so far, immune checkpoint inhibitors plus gemcitabine–cisplatin is the new standard in the first-line therapy in the advanced BTC setting, as also confirmed by the recent European Society for Medical Oncology (ESMO) guidelines (37). However, further studies are needed to identify those patients who may respond better to immune checkpoint inhibitors plus chemotherapy, as well as to determine the best drug type and dose combination, according to the different sites of origin.

## Data availability statement

The data analyzed in this study is subject to the following licenses/restrictions: Types of study to be included. The review will identify manuscripts recording immune checkpoint inhibitors for the treatment of biliary tract cancer, specifically trials in which gemcitabine and cisplatin chemotherapy protocols were compared with standard chemotherapy protocol and immune checkpoint inhibitors in patients with previously untreated unresectable or metastatic biliary tract cancer or with recurrent disease. This systematic and meta-analysis study will include interventional studies, as randomized clinical trials. The present systematic review and meta-analysis was recorded in the PROSPERO register with register no. CRD42023488095. Searches were performed through: Embase, PubMed, Scopus, Web of Science.

## Author contributions

EV: Conceptualization, Data curation, Investigation, Methodology, Resources, Supervision, Visualization, Writing – original draft, Writing – review & editing. AR: Investigation, Resources, Writing – review & editing. LM: Investigation, Methodology, Resources, Writing – review & editing. PN: Supervision, Visualization, Writing – review & editing. TT: Resources, Writing – review & editing. DQ: Resources, Writing – review & editing. SDS: Supervision, Visualization, Writing – review & editing. RM: Supervision, Visualization, Writing – review & editing. NS: Supervision, Visualization, Writing – review & editing. OB: Conceptualization, Data curation, Investigation, Methodology, Resources, Supervision, Validation, Writing – original draft, Writing – review & editing.
